# Prevalence and Impact of Reported Drug Allergies among Rheumatology Patients

**DOI:** 10.3390/diagnostics10110918

**Published:** 2020-11-09

**Authors:** Shirley Chiu Wai Chan, Winnie Wan Yin Yeung, Jane Chi Yan Wong, Ernest Sing Hong Chui, Matthew Shing Him Lee, Ho Yin Chung, Tommy Tsang Cheung, Chak Sing Lau, Philip Hei Li

**Affiliations:** Division of Rheumatology and Clinical Immunology, Department of Medicine, The University of Hong Kong, Queen Mary Hospital, Pokfulam, Hong Kong; shirleychan.ms09@gmail.com (S.C.W.C.); win_as@hotmail.com (W.W.Y.Y.); janewongcy@gmail.com (J.C.Y.W.); ernesto@connect.hku.hk (E.S.H.C.); mattlee233@gmail.com (M.S.H.L.); jameschunghoyin@gmail.com (H.Y.C.); tommyct@gmail.com (T.T.C.); cslau@hku.hk (C.S.L.)

**Keywords:** allergy, drug, hypersensitivity, prevalence, rheumatology

## Abstract

Background: Drug allergies (DA) are immunologically mediated adverse drug reactions and their manifestations depend on a variety of drug- and patient-specific factors. The dysregulated immune system underpinning rheumatological diseases may also lead to an increase in hypersensitivity reactions, including DA. The higher prevalence of reported DA, especially anti-microbials, also restricts the medication repertoire for these already immunocompromised patients. However, few studies have examined the prevalence and impact of reported DA in this group of patients. Methods: Patients with a diagnosis of rheumatoid arthritis (RA), spondyloarthritis (SpA), or systemic lupus erythematosus (SLE) were recruited from the rheumatology clinics in a tertiary referral hospital between 2018 and 2019. Prevalence and clinical outcomes of reported DA among different rheumatological diseases were calculated and compared to a cohort of hospitalized non-rheumatology patients within the same period. Results: A total of 6081 patients (2541 rheumatology patients: 1286 RA, 759 SpA, and 496 SLE; and 3540 controls) were included. DA was more frequently reported among rheumatology patients compared to controls (23.8% vs. 13.8%, *p* < 0.01). Antibiotics and non-steroidal anti-inflammatory drugs (NSAIDs) were the two most commonly reported categories of DA with a prevalence of 12.0% and 5.1%, respectively. Reported antibiotics allergies were more frequent in patients with RA (OR = 1.20, 95% CI = 1.02–1.62, *p* = 0.03) and SLE (OR = 4.69, 95% CI = 3.69–5.95, *p* < 0.01); and associated with increased infection-related admissions among rheumatology patients (OR = 1.79, 95% CI = 1.09–2.95, *p* = 0.02). Among the subgroup of patients referred for allergy testing, 85.7% of beta-lactam antibiotic allergy labels were found to be inaccurate and de-labelled after negative drug provocation testing. Conclusion: The prevalence of reported DA was significantly higher in rheumatology patients. Reported antibiotic allergy was associated with increased rate of infection-related admissions. However, the rate of genuine antibiotic allergy was low. Further studies are needed to guide proper assessment of reported DA and impact of comprehensive allergy testing in this group of patients.

## 1. Introduction

The prevalence of reported drug allergy (DA) is common, affecting around 7% and 14% among the general population and hospitalized patients, respectively [1,2]. Correct diagnosis of genuine DA is important. For example, it is well reported that beta-lactam (BL) antibiotic allergies are common among the general population and come with a myriad of detrimental consequences [2,3,4,5,6,7,8]. However, inaccurate beta-lactam (BL) allergy labels lead to obligatory use of alternative antibiotics which are associated with a multitude of adverse clinical consequences including increased healthcare costs, intensive care admissions, longer hospital stays, and death; as well as increased risks of multi-drug resistant organisms [9,10,11,12,13]. Unfortunately, the majority of self- or physician-reported DA (especially with BL), are likely inaccurate. In both Western and Asian studies, we previously found that only around 10% of patients with reported BL allergies were found to be truly allergic after testing [1,2,14].

The pathophysiology and manifestations of genuine DA depends on a variety of drug and patient factors, including concomitant disease states such as rheumatological diseases [15,16]. Higher rates of DA have previously been reported among rheumatology patients, especially in patients with systemic lupus erythematosus (SLE) [17,18,19,20]. However, literature is scarce and very little is known about the prevalence or impact of DA, especially among other rheumatological diseases.

Patients with rheumatological diseases are often immunosuppressed and are more susceptible to infectious complications. We postulate that having inaccurate DA labels in this population will lead to even greater consequences. The dysregulated immune system underpinning rheumatological diseases may also lead to an increase in hypersensitivity reactions. Rheumatology patients are predisposed to be exposed and sensitized to various immunosuppressants and immunomodulatory medications. However, the burden and adverse consequences of reported DA among rheumatology patients remains to be investigated. To elucidate these areas of uncertainty, this study aims to assess the prevalence and impact of reported DA among rheumatology patients with rheumatoid arthritis (RA), spondyloarthritis (SpA), and SLE.

## 2. Methods

All patients with a diagnosis of RA, SpA, or SLE were recruited from the rheumatology clinics of Queen Mary Hospital (QMH) between 2018 and 2019. Baseline demographics, clinical parameters, and details of physician-reported DA were obtained by medical record review. Prevalence and categories of reported DA among patients with rheumatological diseases (“rheumatology patients”) were calculated and compared to a cohort of hospitalized patients admitted to the acute general medical wards of QMH without a diagnosis of RA, SpA, SLE, or other connective tissue diseases within the same period (“controls”). Number of hospital admissions and principal diagnoses of admissions (categorized into infection-related or not) were recorded. Hospital admissions with incomplete records or deemed not related to infections (after medical record review by 2 independent reviewers) were excluded. Frequency of infection-related admissions in rheumatology patients with or without DA were compared.

All rheumatology patients with infection-related admissions and reported DA to BL underwent formal BL allergy testing, including skin testing (ST) with or without drug provocation testing (DPT). All patients had ST performed with benzylpenicilloyl polylysine (0.04 mg/mL), sodium benzylpenilloate (0.5 mg/mL), benzylpenicillin (10,000 UI/mL), amoxicillin (25 mg/mL), and/or index BL as per European Network and European Academy of Allergy and Clinical Immunology recommendations [21]. Patients with positive ST were deemed genuinely allergic and did not proceed with subsequent DPT. Patients with negative ST proceeded with DPT with the same BL as per their index reaction (and if the index BL was unknown, DPT was performed with the BL most clinically relevant for the patient—in most cases, amoxicillin).

QMH is the only public hospital in the Hong Kong West Cluster providing acute medical admissions via its Accident and Emergency Department. Patients therefore likely represent the general population of Hong Kong requiring acute medical care during the study period. Informed consent was waived, and data extraction was approved by the Institutional Review Board of the University of Hong Kong/ Hospital Authority Hong Kong West Cluster (Reference number: UW 18–669, approved 19 December 2018).

## 3. Statistical Analysis

Baseline characteristics of patients were compared using Student’s t-test for continuous variables and the χ2 test for categorical variables between patients with and without reported drug allergy (univariate analysis). The prevalence of DA between rheumatology patients and controls were compared with adjustment for age and sex. Logistic regression analysis (multivariate analysis) was performed to evaluate predictors of infection-related admissions using demographics and DA information. All statistics were performed using the International Business Machines Corporation Statistical Package for the Social Sciences (IBM SPSS) package 26.0.

## 4. Results

A total of 2541 unique rheumatology patients (1286 RA, 759 SpA, and 496 SLE) and 3540 controls were recruited during the study period. The baseline characteristics and demographics of all recruited patients are shown in Table 1.

### 4.1. Prevalence of Reported DA Was Significantly Higher among Rheumatology Patients, Affecting 40.1% of SLE Patients and 22.3% of RA Patients

**Figure 1:**
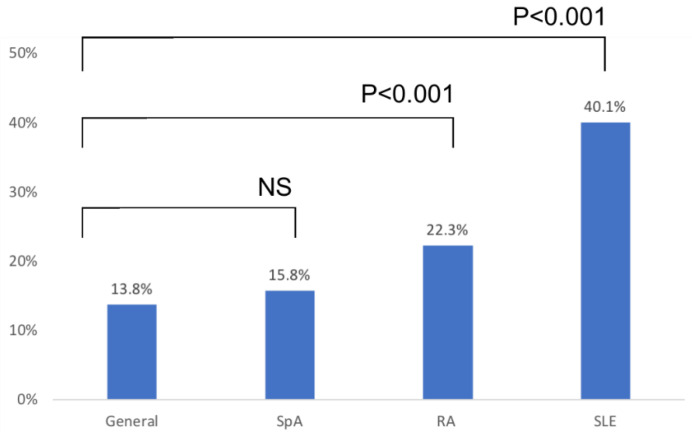
Prevalence of reported drug allergy among different rheumatological diseases.

Overall, reported DA was significantly more frequent among rheumatology patients compared with controls (OR = 1.96, 95% CI = 1.72–2.24, *p* < 0.01). When analysing individual rheumatological diseases, this difference was most significant among patients with SLE (OR = 4.20, 95% CI = 3.43–5.15, *p* < 0.01), followed by RA (OR = 1.81, 95% CI = 1.30–2.12, *p* < 0.01) patients. However, there was no significant difference between patients with SpA and controls (Figure 1).

### 4.2. Antibiotics (Especially Beta-Lactams) and NSAID Were the Most Frequently Reported DA among Rheumatology Patients, and Significantly More Than Controls

**Figure 2:**
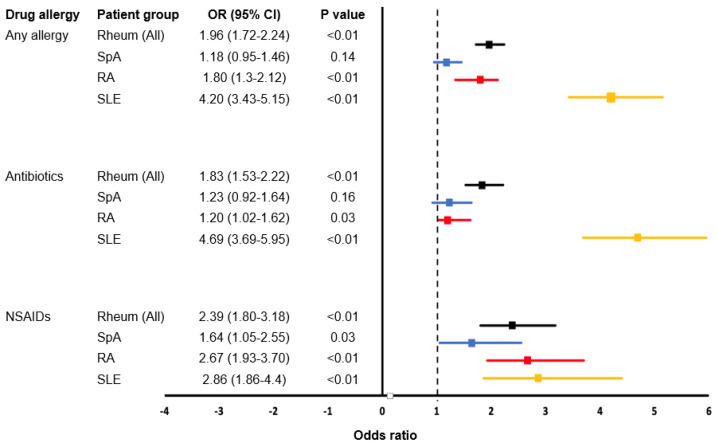
Age- and sex- adjusted odds ratios of DA (by disease and drug categories) compared to controls.

Among rheumatology patients, the most commonly reported DA were to antibiotics (12.0%), especially BL antibiotics (7.6%), followed by non-steroidal anti-inflammatory drugs (NSAIDs; 5.1%), disease-modifying antirheumatic drugs/immunosuppressants (4.4%), cardiovascular medications (2.6%), and intravenous radiocontrast (1.3%; Table 2 and Supplementary Figure S1). Using multivariate analysis to adjust for age and sex, reported antibiotic DA was more frequent among rheumatology patients compared with controls, especially among patients with RA (OR = 1.20, 95% CI 1.02–1.62, *p* = 0.03) and SLE (OR = 4.29, 95% CI = 3.23–5.69, *p* < 0.01). Reported DA to NSAIDs was more frequent across all rheumatology patients compared with general patients. Age and sex-adjusted odds ratios of DA by disease and drug classes are shown in Figure 2.

### 4.3. Reported Antibiotic DA Was Independently Associated with Increased Rate of Infection-Related Admissions

Within the study period, 4.1% of all rheumatology patients were hospitalized due to infections. Univariate analysis revealed that age (62.0 ± 14.5 vs. 54.9 ± 13.7 years, *p* < 0.01) and reported antibiotic allergy (20.4% vs. 11.7%, *p* = 0.01) were significantly associated with infection-related admissions (Table 3). The association between reported antibiotic allergy and infection-related admissions remained significant in multivariate analysis (OR = 1.79; 95% CI = 1.09–2.95, *p* = 0.02).

### 4.4. Drug Allergy Testing Was Useful, and Genuine BL Allergy Was Infrequent among Rheumatology Patients

There were 13 rheumatology patients with infection-related admissions and reported DA to BL. Two patients succumbed during their infection-related admission before evaluation of their reported DA. The remaining 11 patients were referred to undergo formal BL allergy testing. Five patients with seven reported BL allergies (with two patients having multiple reported BL allergies) agreed and were able to complete BL allergy evaluation. Clinical histories and results of their BL allergy workup are shown in Supplementary Table S1. The majority (85.7%, 6/7) of reported BL allergy labels were proven to be inaccurate and removed after confirmation with DPT.

## 5. Discussion

Adverse drug reactions (ADR) are defined as a “response to a drug which is noxious and unintended, which occurs at doses normally used in man” which can occur via a variety of different immunological or non-immunological mechanisms [22]. DA is defined as an ADR which is immunological mediated, and can be classified according to the immunologic mechanism described in Gell and Coombs classification of hypersensitivity reactions, with the vast majority presumed to be either IgE- (type I) or T cell-mediated (type IV) hypersensitivity reactions (HSR) [23]. Rheumatological diseases and allergic conditions are both characterized by immune dysregulation, which might be one of the factors contributing to the prevalence of DA in this group of patients.

The dysregulated immune system is responsible for the inappropriate response towards autoantigens in rheumatological diseases, and to exogenous factors in allergic disorders including DA. It has been reported that allergic conditions are more common in patients with rheumatological diseases, such as RA, SpA, and SLE [24,25,26]. Studies examining the links between allergic airway disease and rheumatological diseases are more prominent, with multiple mechanisms proposed to account for the link, including genetic predisposition, environmental factors, and immune regulations involving the interplay between Th1 and Th2 immunity, mast cell responses, and role of regulatory B cells [27,28]. Even though it has been observed that DA is more common among patients with rheumatological diseases, little is known so far regarding the potential immunopathogenic pathways involved.

Our study showed that the prevalence of reported DA was significantly higher among SLE and RA patients in comparison to hospitalized non-rheumatology, but not SpA. Susceptibility to more DA (both genuine DA and inaccurate DA labelling) in rheumatology patients could be due to various factors. Previous studies suggested other susceptible factors among rheumatology patients, such as gender, genetic polymorphism, and viral infections [29]. DA is more frequently reported in females, and genetic and hormonal factors have been proposed to play a role in this phenomenon [30]. Similarly, rheumatological diseases also have a predilection to affect female patients, especially SLE and RA. Although the proportion of female patients were much high than in the control group (70.1 vs. 53.4%), the risk of DA was still higher among patients with rheumatological diseases, adjusted by age and sex, implying that other factors are also involved.

We postulate additional mechanisms, such as with the example of immediate-type DA. Immediate type (type I) HSR are classically thought to be IgE-mediated. Another predominantly IgE-autoantibody (or IgG-autoantibody to IgE and FcεRI) mediated immunological condition which manifests very similarly is chronic spontaneous urticaria (CSU), with an autoimmune subset being increasingly recognized [31,32]. It is known that various autoimmune diseases, such as SLE and RA, are also prone to development of CSU [33]. One postulation is the development of autoantibodies to FcεRI or IgE on both mast cells and basophils. Furthermore, the role of IgE is likely shared amongst various autoimmune diseases. In fact, a study demonstrated the increased pathogenic role of IgE autoantibodies in SLE patients, in particular, increased IgE to anti-dsDNA, anti-Sm, anti-Ro/SSA, and anti-La/SSB, which had a positive correlation with active disease [34]. The propensity for production of IgE autoantibodies may have a role in production of drug specific IgE. On the contrary, the pathogenesis of SpA leans less towards B cell dysregulation, and we found SpA patients had similar reported DA compared to controls.

Furthermore, rheumatology patients are more likely to have concomitant CSU, and urticaria flares are known to occur more frequently during infective episodes when new medications (especially antimicrobials) are prescribed. Similarly, NSAIDs use is frequent among rheumatology patients and may exacerbate urticaria in those with concomitant CSU (NSAIDs-exacerbated cutaneous disease). During episodes of acute urticarial flares, physicians may often wrongly label DA as a culprit of urticaria or angioedema, leading to an overall increased frequency of reported DA among rheumatology patients. Differentiating between a genuine allergy and an ADR is a diagnostic challenge [35], especially in rheumatology patients who may exhibit symptoms that mimic allergic reactions such as skin rash. Education and awareness of the complexity of diagnosing DA in rheumatology patients are important, and interdisciplinary collaboration between rheumatologist and allergist are imperative.

Owing to the lack of allergists and DA testing services in Hong Kong, the waiting time for proper drug allergy workup can take months or years [36]. In our study, more than 85% of reported BL allergy in rheumatology patients previously admitted for infections were confirmed to be inaccurate by DPT. Only one patient, labelled with both an ampicillin and cloxacillin allergy after an index reaction of angioedema, was found to be genuinely allergic. Subsequent ST confirmed an IgE-mediated selective hypersensitivity to cloxacillin, and tolerance to amoxicillin was demonstrated by a negative DPT. Given this very low rate of genuine DA and our novel finding that reported antibiotic DA labels are associated with increased hospitalization rates among rheumatology patients, many infection-related hospital admissions may be avoided if adequate allergy testing services were readily available.

There are several limitations to our study. First, because of its observational nature, there were several factors that we were not able to be obtained from the computer records alone. For example, whether the DA was suspected to be immediate or delayed-type was often unclear or missing. Risk factors for DA labels, such as timing of the index reaction, are also missing but important independent variables. Additionally, other comorbidities, such as cardiovascular diseases, non-rheumatological pain syndromes, atopic diseases, and non-rheumatological immunosuppressive factors, were not included. This highlights the importance of dedicated and prospective studies in the future. Second, our control group consisted of hospitalized patients, and comparison between general rheumatology patients can be difficult. However, we know that the prevalence of reported DA is higher among hospitalized patients compared with the general population [1,2]. We would therefore expect a bias (if any) toward DA among the control cohort when compared to rheumatology patients. Despite this potential bias, rheumatology patients still had a significantly higher prevalence of DA labels, which further demonstrates the overwhelming higher prevalence of reported DA in this susceptible cohort. Third, our study mainly focused on antibiotic allergies. In particular, we focused on BL as it was the most common DA label reported. Additionally, skin tests for penicillin are well validated unlike other medications such as non-BL antibiotics and NSAIDs. Future studies looking at other allergies will be useful to get a broader sense of the burden DA have in this cohort. For example, workup for genuine NSAID allergy could be useful, especially in SpA patients where other non-biologic disease modifying agents remain limited. Similarly, proper evaluation of biologics allergy in the literature is also scarce and warrant studies to guide diagnostic algorithms in this special population [37,38].

In conclusion, our study sheds light on the burden and impact of DA in rheumatological diseases. The prevalence of reported DA was significantly higher in rheumatology patients, highlighting the importance for further research to understand the underlying mechanisms involved. Reported antibiotic allergy was also associated with increased rate of infection-related admissions among rheumatology patients, although the rate of genuine BL antibiotic allergy was low. Further studies are needed to guide proper assessment of reported DA and impact of comprehensive allergy testing in this group of patients.

## Figures and Tables

**Table 1:** Baseline characteristics and demographics of rheumatology patients (N = 2541) and controls (N = 3540).

All Patients (N = 6081)				
	Total	No drug allergy	Drug allergy	*p* value
Number	6081	4988 (82.0%)	1093 (18.0%)	
Age (years)	64.6 ± 18.0	64.7 ± 18.3	63.7 ± 16.0	0.06
Male	2393 (39.4%)	2089 (41.9%)	304 (27.8%)	<0.01
Controls (N = 3540)				
	Total	No drug allergy	Drug allergy	*p* value
Number	3540	3053 (86.2%)	487 (13.8%)	
Age (years)	71.3 ± 17.6	71.4 ± 17.7	71.0 ± 17.0	0.65
Male	1649 (46.6%)	1458 (47.8%)	191 (39.2%)	<0.01
Rheumatology patients (N = 2541)				
	Total	No drug allergy	Drug allergy	*p* value
Number	2541	1935 (76.2%)	606 (23.8%)	
Age (years)	55.2 ± 13.8	54.3 ± 14.1	57.9 ± 12.5	<0.01
Male	744 (29.3%)	631 (32.6%)	113 (18.6%)	<0.01
Rheumatoid arthritis (N = 1286)				
	Total	No drug allergy	Drug allergy	*p* value
Number	1286	999 (77.7%)	287 (22.3%)	
Age (years)	58.9 ± 12.1	58.2 ± 12.2	61.3 ± 11.8	<0.01
Male	228 (17.7%)	189 (18.9%)	39 (13.6%)	0.04
Spondyloarthritis (N = 759)				
	Total	No drug allergy	Drug allergy	*p* value
Number	759	639 (84.2%)	120 (15.8%)	
Age (years)	49.5 ± 15.0	48.8 ± 15.0	53.3 ± 14.2	<0.01
Male	473 (62.3%)	416 (65.1%)	57 (47.5%)	<0.01
Systemic lupus erythematosus (N = 496)				
	Total	No drug allergy	Drug allergy	*p* value
Number	496	297 (59.9%)	199 (40.1%)	
Age (years)	54.1 ± 12.7	53.0 ± 13.6	55.7 ± 11.1	0.01
Male	43 (8.7%)	17 (8.5%)	26 (8.8%)	0.94

**Table 2:** Frequency of different classes of reported drug allergies (DA).

	Rheumatology Patients (N = 2541)					Controls (N = 3540)	
	RA	SLE	SpA	Total	%	Total	%
Any antibiotics	113	129	64	306	12	247	7
Beta-lactam antibiotics	69	82	43	194	7.6	170	4.8
Non-steroidal anti-inflammatory drugs	73	30	27	130	5.1	78	2.2
Disease-modifying antirheumatic drugs/immunosuppressants	79	19	15	113	4.4	0	0
Cardiovascular drugs	42	19	4	65	2.6	59	1.7
Intravenous contrast	14	11	8	33	1.3	41	1.2
Other analgesics	16	6	8	30	1.2	12	0.3
Allopurinol	3	4	1	8	0.3	17	0.5
Anti-fungal	4	2	2	8	0.3	4	0.1
Anti-virals	3	4	0	7	0.3	3	0.1

**Table 3:** Association analysis between clinical features and infection-related hospitalization.

Rheumatology Patients (N = 2541)						
			Univariate		Multivariate	
	Admission	No Admission	OR (95%CI)	*p* Value	OR (95%CI)	*p* Value
Number of patients	103 (4.1%)	2438 (95.9%)	------	------	------	------
Age	62.0 ± 14.5	54.9 ± 13.7	------	<0.01	1.04 (1.03–1.06)	<0.01
Male	33 (32.0%)	711 (29.2%)	1.15 (0.75–1.75)	0.53	------	------
RA	52 (50.5%)	1234 (50.6%)	1.00 (0.67–1.48)	0.98	------	------
SpA	26 (25.2%)	733 (30.1%)	0.79 (0.50–1.24)	0.30	------	------
SLE	25 (24.3%)	471 (19.3%)	1.34 (0.84–2.12)	0.21	------	------
Reported DA						
Antibiotics	21 (20.4%)	285 (11.7%)	1.94 (1.18–3.17)	0.01	1.79 (1.09–2.95)	0.02
NSAIDs	9 (8.7%)	121 (5.0%)	1.83 (0.90–3.72)	0.09	------	------

RA = rheumatoid arthritis, SpA = spondyloarthritis; SLE = systemic lupus erythematosus; NSAIDs = non-steroidal anti-inflammatory drugs.

## Supplementary Materials

The following are available online at https://www.mdpi.com/2075-4418/10/11/918/s1, Figure S1: Frequency of reported DA in 2541 rheumatology patients; Table S1: Allergy histories and investigation results of patients who completed beta-lactam allergy testing.
