# Supplementary material for: Prevalence and Impact of Reported Drug Allergies among Rheumatology Patients
Source: Diagnostics (Basel). 2020 Nov 9;10(11):918. doi: 10.3390/diagnostics10110918 (PMC7695245; doi:10.3390/diagnostics10110918)
Supplement: Supplementary file 1 [file diagnostics-10-00918-s001.pdf]

Supplementary Table S1: Frequency of different classes of reported DA.

	Rheumatology patients (N=2541)					Controls (N=3540)	
	RA	SLE	SpA	Total	%	Total	%
Any antibiotics	113	129	64	306	12	247	7
- Beta-lactam antibiotics	69	82	43	194	7.6	170	4.8
Non-steroidal anti-inflammatory drugs	73	30	27	130	5.1	78	2.2
Disease-modifying antirheumatic drugs/immunosuppressants	79	19	15	113	4.4	0	0
Cardiovascular drugs	42	19	4	65	2.6	59	1.7
Intravenous contrast	14	11	8	33	1.3	41	1.2
Other analgesics	16	6	8	30	1.2	12	0.3
Allopurinol	3	4	1	8	0.3	17	0.5
Anti-fungal	4	2	2	8	0.3	4	0.1
Anti-virals	3	4	0	7	0.3	3	0.1

Supplementary Table S2: Allergy histories and investigation results of patients who completed beta-lactam allergy testing.

	Age	Sex	Diagnosis	DMARDs/IST	Reported DA	Other DA	Index Reactions	Allergy investigation results
1	74	F	RA	Nil	"Penicillins" (unknown index)	Aspirin Betahistine Cinnarizine	Shortness of breath, syncope	Negative skin tests to PPL, MDM, BP and amoxicillin. Negative DPT to amoxicillin
2	45	F	RA	Methotrexate 15mg weekly, sulfasalazine 1g BD	Amoxicillin Cloxacillin	Mefenamic acid	Angioedema	Negative skin tests to PPL, MDM, BP and amoxicillin. Positive skin test to cloxacillin Negative DPT to amoxicillin
3	79	M	RA	Tofacitinib 5mg BD	Amoxicillin Meropenem	Nitrofurantoin Vancomycin Penicillamine	Unknown	Negative skin tests to PPL, MDM, BP, amoxicillin and meropenem. Negative DPT to amoxicillin and meropenem
4	75	M	SpA	Nil	Amoxicillin	Phensedyl	Rash	Negative skin tests to PPL, MDM, BP and amoxicillin. Negative DPT to amoxicillin
5	64	M	SLE	Nil	"Penicillins" (unknown index)	Nil	Rash	Negative skin tests to PPL, MDM, BP and amoxicillin. Negative DPT to amoxicillin
